# Prognostic value of ferroptosis‐related genes in patients with lung adenocarcinoma

**DOI:** 10.1111/1759-7714.13998

**Published:** 2021-05-12

**Authors:** Guangsheng Zhu, Hua Huang, Songlin Xu, Ruifeng Shi, Zhouyong Gao, Xi Lei, Shuai Zhu, Ning Zhou, Lingling Zu, Ramon A. De Mello, Jun Chen, Song Xu

**Affiliations:** ^1^ Department of Lung Cancer Surgery Tianjin Medical University General Hospital Tianjin China; ^2^ Tianjin Key Laboratory of Lung Cancer Metastasis and Tumor Microenvironment Lung Cancer Institute, Tianjin Medical University General Hospital Tianjin China; ^3^ Tianjin Medical University Tianjin China; ^4^ Escola Paulista de Medicina Federal University of Sao Paulo Sao Paulo Brazil

**Keywords:** ferroptosis, ferroptosis‐related genes, LUAD, lung adenocarcinoma

## Abstract

**Background:**

The prevalence of lung adenocarcinomas (LUADs) has dramatically increased in recent decades. Ferroptosis is a process of iron‐dependent regulatory cell death. It is still unclear whether the expression of ferroptosis‐related genes (FRGs) is involved in the pathogenesis and survival of patients with LUAD.

**Methods:**

We retrieved LUAD data from The Cancer Genome Atlas (TCGA) and Gene Expression Omnibus (GEO) databases and used LASSO Cox regression analysis to select the gene signature suitable for modeling. The risk score was calculated according to the model, and the patients were divided into high‐ and low‐risk groups according to the median risk score. Functional enrichment analysis was carried out by this group, and a model for predicting clinical prognosis was established by combining this group with clinical factors.

**Results:**

Gene set enrichment analysis (GSEA) and single‐sample gene set enrichment analysis (ssGSEA) analysis showed that there were several immune‐related pathways and immune infiltration differences between high‐ and low‐risk groups. A prognostic model integrating 10 ferroptosis‐related genes (FR‐DEGs), and clinical factors were constructed and validated in an external cohort.

**Conclusions:**

The FR‐DEGs signature was related to immune infiltration, and a model based on FR‐DEGs and clinical factors was established to predict the prognosis of patients with LUAD.

## Graphical abstract

FR‐DEGs signature has a high correlation with the clinical characteristics and immune microenvironment of lung adenocarcinoma patients. The prognosis prediction model of LUAD patients based on FR‐DEGs and clinical characteristics can accurately predict the prognosis of lung adenocarcinoma patients.

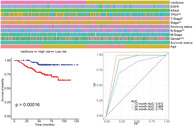

## INTRODUCTION

Lung cancer (LC) is one of the most frequently reported malignancies worldwide and the most common cause of global cancer‐associated mortality, with over a million people succumbing each year.^1^Based on the histology, LC is divided into two main subtypes: small cell lung carcinoma and non‐small cell lung carcinoma (NSCLC), accounting for 15% and 85% of all cases, respectively.^2^ Lung adenocarcinoma (LUAD) has increased in prevalence compared to other subtypes of lung cancer.^3^ Increasing numbers of non‐ and never‐smokers are developing LUADs.^4^ As a newly discovered type of programmed cell death, ferroptosis results from the accumulation of iron‐dependent lipid hydroperoxides and leads to cytological changes; the features and mechanisms of ferroptosis are different from those of typical cell death processes, such as apoptosis.^5^ It is closely related to the metabolism of amino acids, iron, and polyunsaturated fatty acids, and the biosynthesis of glutathione, phospholipids, NADPH, and coenzyme Q10.^6^, ^7^

Recently, ferroptosis has been found to have great potential in cancer treatment, especially in tumors resistant to traditional therapy.^8^, ^9^ Increasing evidence has shown that numerous tumor cells, including ovarian cancer cells^10^ and hepatocellular carcinoma cells,^11^ are sensitive to it. Furthermore, previous studies have shown that ferroptosis suppresses tumor growth and kills tumor cells^12^ and plays an important role in cancers such as renal cell carcinomas and adrenocortical carcinoma cells.^12^, ^13^ However, the relationship between ferroptosis and LUAD patient prognoses has yet to be elucidated. In this study, we analyzed the differential expression of ferroptosis‐related genes (FRGs) in LUAD patients from a public database to identify the enriched pathways and their biological functions and constructed an FR‐DEGs and clinical factors‐based model for LUAD prognosis evaluation. Previous evidence indicates that CD8+ T cells enhance ferroptosis by downregulating SLC3A2 and SLC7A11, and the induction of ferroptosis contributes to the antitumor efficacy of immunotherapy, suggesting that the immune system might function through ferroptosis.^14^ Moreover, ferroptotic cancer cells might release signals such as oxidized lipid mediators to affect antitumor immunity.^15^ which indicates that FRGs could become a promising prognostic marker of immunotherapy in LUAD. Although many different prognostic biomarkers and models have been established in patients with LUAD, few studies have focused on the comprehensive status of the immune pathway.

## METHODS

### Data source

RNA sequencing (RNA‐seq) data and corresponding clinical information of 535 patients with LUAD (including 59 normal lung tissues and 535 tumor tissues) patients were downloaded from The Cancer Genome Atlas (https://portal.gdc.cancer.gov/repository)(TCGA cohort). Gene microarray data and clinical information of 226 tumor samples were obtained from the Gene Expression Omnibus (GEO) dataset (https://www.ncbi.nlm.nih.gov/geo/query/acc.cgi?acc=GSE31210) (GEO cohort).^16^ Patients who met the following selection criteria were included: (i) Histologically diagnosed with LUAD, (ii) available gene expression data, and (iii) available survival information. The baseline characteristics of patients in these two cohorts are summarized in Table 1. In this study, we chose the TCGA cohort as training set, and GEO cohort as verification set.

**TABLE 1:** Clinical characteristics of the TCGA and GEO cohorts

Characteristic		TCGA cohort	GEO cohort
Age	<60	151 (28%)	108 (48%)
	≥60	330 (62%)	118 (52%)
Gender	Man	230 (43%)	105 (46%)
	Woman	270 (50%)	121 (54%)
Smoking status	Ever	414 (77%)	111 (49%)
	Never	72 (13%)	115 (51%)
Stage	I–II	390 (73%)	226 (100%)
	III–IV	108 (20%)	0

### Model establishment and validation of prognostic ferroptosis‐related gene signature

A total of 60 FRGs were retrieved from the previous literature and are provided in Table S1. We performed the following process to establish the immune signature. The “limma” R package was first used to identify the differentially expressed 60 FRGs between tumor tissues and adjacent nontumorous tissues with a false discovery rate (FDR) < 0.05 in the TCGA cohort. Univariate Cox analysis of overall survival (OS) was performed to screen for FRGs with prognostic values. *p*‐values < 0.05 were considered statistically signifcant. Least absolute shrinkage and selection operator (LASSO) Cox regression analysis was then applied to construct a prognostic model.^17^, ^18^ LASSO Cox regression was calculated using the “glmnet” package in R software, and the ideal coefficients were evaluated according to the partial likelihood deviance with tenfold cross validation. The risk scores of the patients were calculated according to the normalized expression level of each gene and its corresponding regression coefficients. The formula was established as follows: score = esum (each gene's expression × corresponding coefficient). The patients were stratified into high‐ and low‐risk groups based on the median value of the risk score. Based on the expression of genes in the signature, principal component analysis (PCA) was carried out with the “prcomp” function of the “stats” R package. In addition, t‐SNE were performed to explore the distribution of different groups using the “Rtsne” R package. The “survivalROC” R package was used to conduct time‐dependent receiver operating characteristic (ROC) curve analyses to evaluate the predictive power of the gene signature.

### Functional enrichment analysis

The “clusterProfiler” R package was utilized to conduct Gene Set Enrichment Analysis (GSEA), analysis of gene ontology (GO), and Kyoto Encyclopedia of Genes and Genomes (KEGG) between the high‐ and low‐risk groups. FDR < 0.25 was considered statistically significant. The infiltrating score of 16 immune cells and the activity of 13 immune‐related pathways were calculated using single‐sample gene set enrichment analysis (ssGSEA)^19^ in the “gsva” R package. The annotated gene set file is provided in Table S2.

### Statistical analysis

The Wilcoxon test was used to compare gene expression between tumor tissues and adjacent nontumorous tissues. Differences in proportions were compared using the chi‐square test. The Wilcoxon test was used to compare the ssGSEA scores of immune cells or pathways between the high‐ and low‐risk groups. The OS between different groups was compared using Kaplan–Meier analysis with the log‐rank test. Univariate and multivariate Cox regression analyses were performed to identify independent predictors of OS. All statistical analyses were performed using R software (Version 4.0.2). A *p*‐value of less than 0.05 was considered statistically significant, and all *p*‐values were two‐tailed.

## RESULTS

**FIGURE 1:**
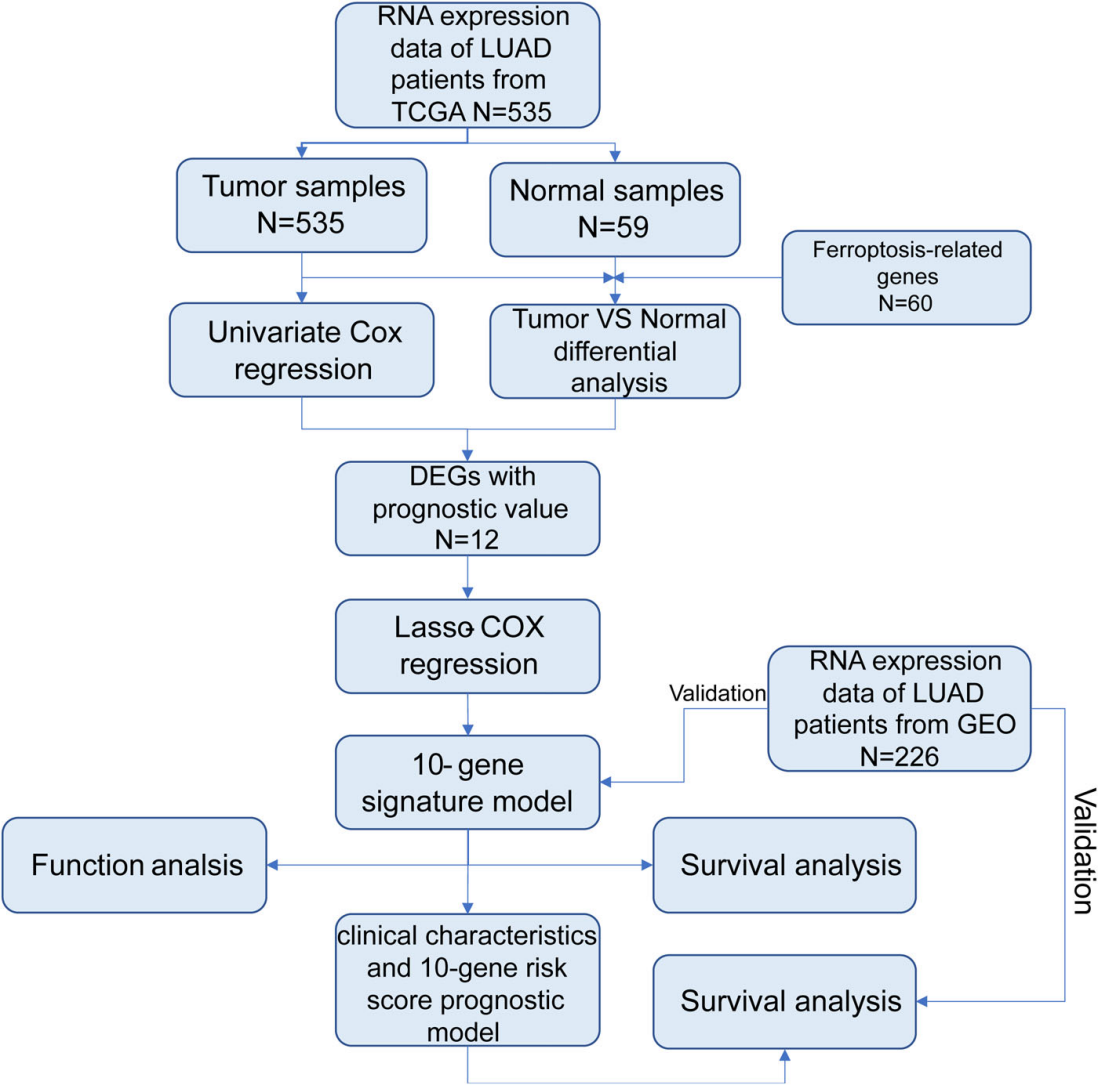
Flow chart of the study

The flow chart of this study is shown in Figure *1*. A total of 535 patients with LUAD from the TCGA‐LUAD cohort and 226 patients with LUAD from the GEO cohort were finally enrolled. The detailed clinical characteristics of these patients are summarized in Table *1*.

### Identification of FR‐DEGs and their prognostic value in the TCGA cohort

**FIGURE 2:**
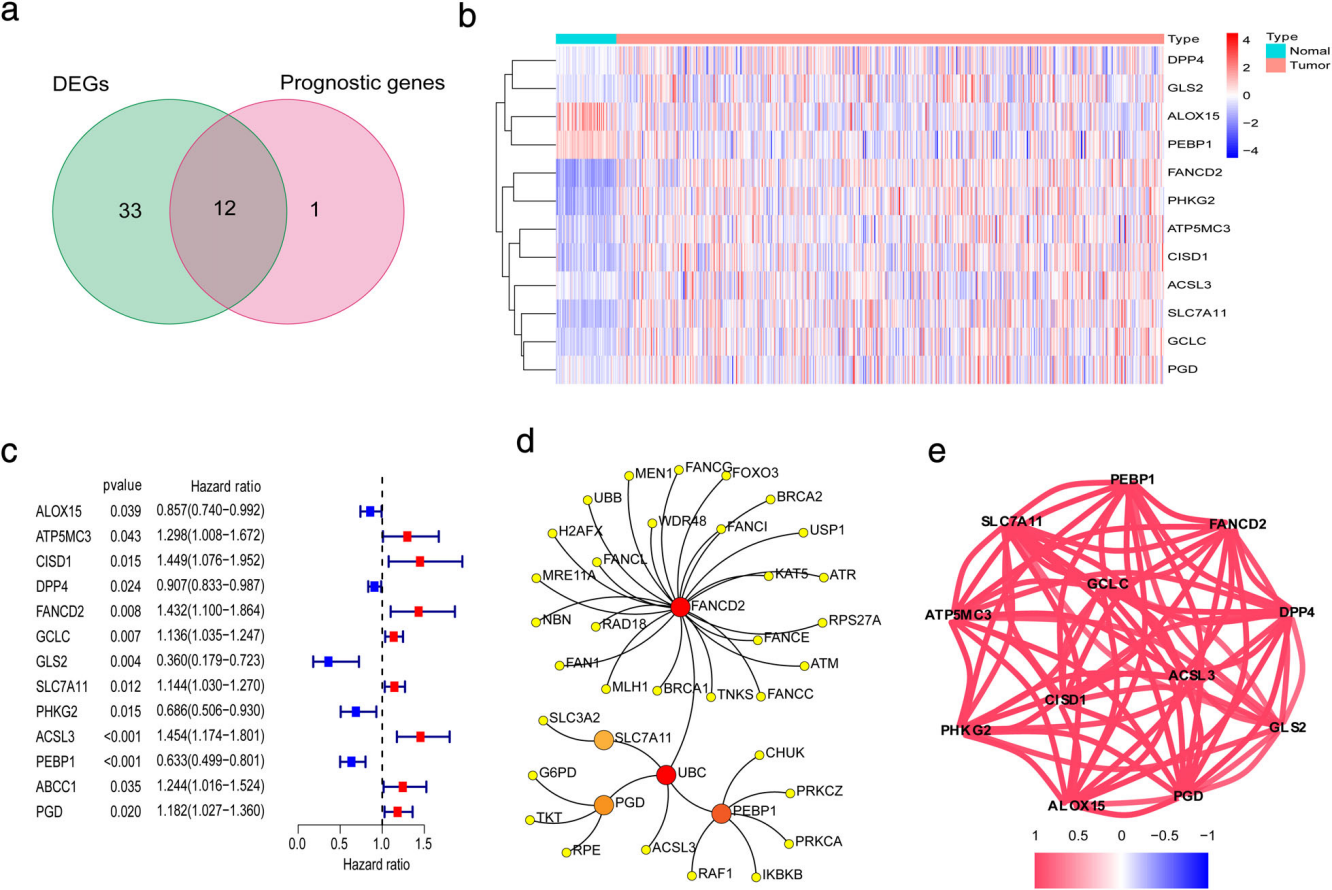
Identification of the candidate ferroptosis‐related genes in The Cancer Genome Atlas (TCGA) cohort. (a) Venn diagram which identified differentially expressed genes between tumor and adjacent normal tissue that were correlated with overall survival (OS). (b) The expression of 12 ferroptosis‐related genes (FRGs) in lung adenocarcinoma (LUAD) patient samples is shown. The upregulated FRGs are indicated in red and downregulated FRGs in blue. (c) Univariate Cox regression analysis results show the association between gene expression and OS. (d) The PPI network downloaded from the STRING database indicated the interactions among the candidate genes. (e) The correlation network of candidate genes. The correlation coefficients are represented by different colors

Most of the FRGs (45/60, 75%) were differentially expressed between tumor tissues and adjacent nontumorous tissues, and 12 were correlated with OS in the univariate Cox regression analysis (Figure *2*(a)). A total of 12 prognostic ferroptosis‐related DEGs were preserved (all FDR < 0.05, Figure *2*(b), (c)). The interaction network among these genes indicated that FANCD2 and PEBP1 were the hub genes (Figure *2*(d)). The correlation between these genes is shown in Figure *2*(e).

### Establishment and validation of prognostic model

**FIGURE 3:**
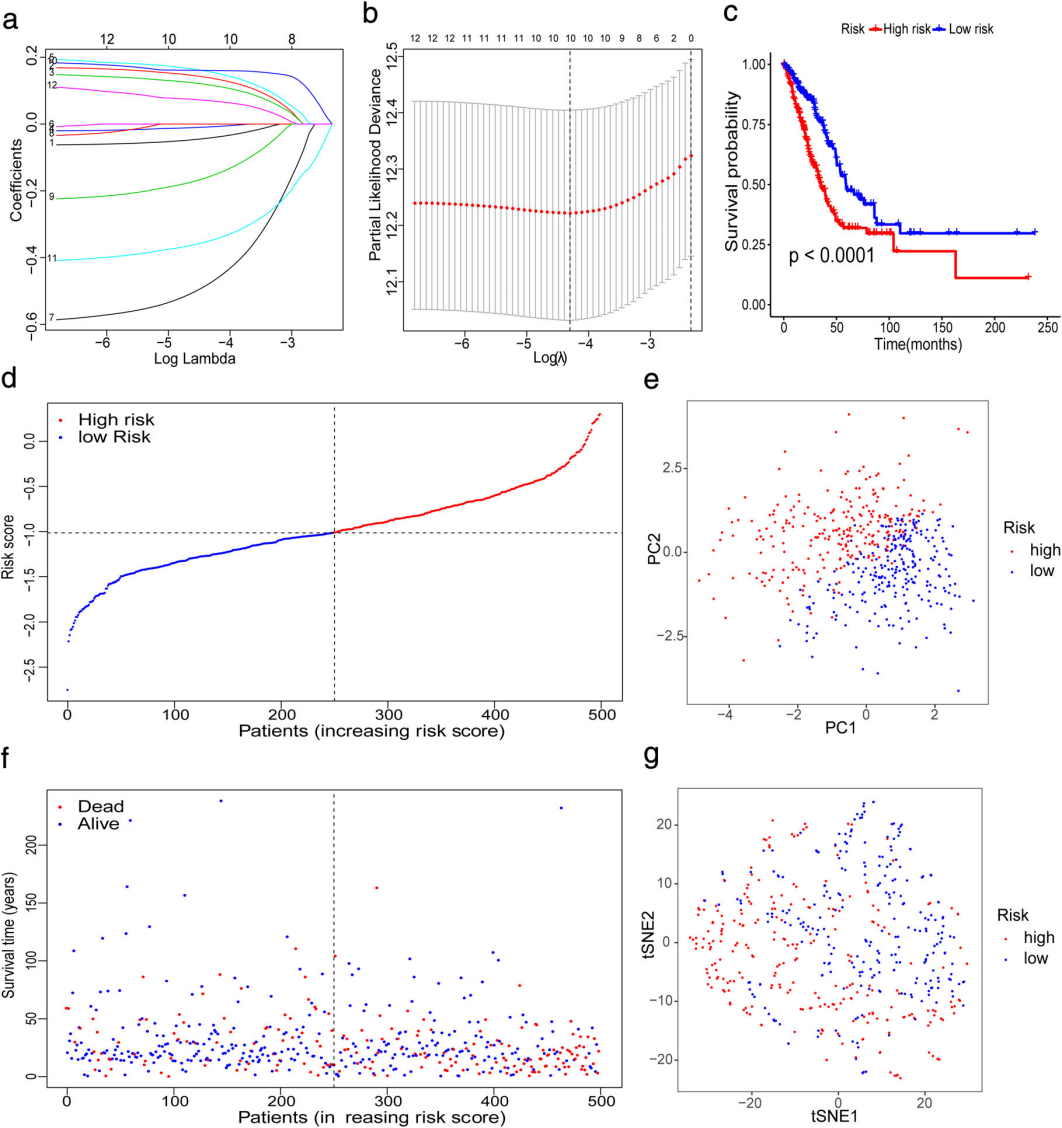
Prognostic analysis of the 10‐gene signature model in The Cancer Genome Atlas (TCGA) cohort. (a) LASSO coefficient profiles of the expression of 12 candidate genes. (b) Selection of the penalty parameter (λ) in the LASSO model via 10‐fold cross‐validation. The dotted vertical lines are plotted at the optimal values following the minimum criteria (left) and “one standard error” criteria (right). (c) Kaplan–Meier curves for the overall survival (OS) of patients in the high‐ and low‐risk groups in the TCGA cohort. (d) The distribution and median value of the risk scores in the TCGA cohort. (e) Principal component analysis (PCA) plot of the International Cancer Genome Consortium (ICGC). (f) The distributions of OS status, OS and risk score (g) t‐SNE analysis of the ICGC cohort

**FIGURE 4:**
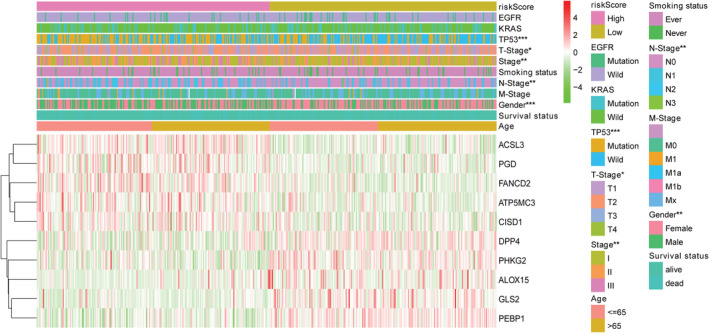
The heatmap shows the profiles of the expression of survival model ferroptosis‐related genes (FRGs) and clinicopathological features in low‐ and high‐risk lung adenocarcinoma (LUAD) patients

LASSO Cox regression analysis was applied to establish a prognostic model using the expression profile of the 12 genes mentioned above. A 10‐gene signature was identified based on the optimal value of λ (Figure *3*(a), (b)). The patients were stratified into high‐ or low‐risk groups according to the median cutoff value (Figure *3*(d)). The higher risk group was significantly associated with higher tumor stage, TP53 mutation, sex, and advanced tumor node metastasis (TNM) stage in the TCGA cohort (Figure *4*). PCA and t‐SNE analysis indicated that the patients in the different risk groups were distributed in two directions (Figure *3*(e), (g)). As shown in Figure *3*(f), patients with high risk had a higher probability of death earlier than those with low risk. The Kaplan–Meier curve consistently indicated that patients in the high‐risk group had a significantly worse OS than their low‐risk counterparts (Figure *3*(c), *p* < 0.0001).

**FIGURE 5:**
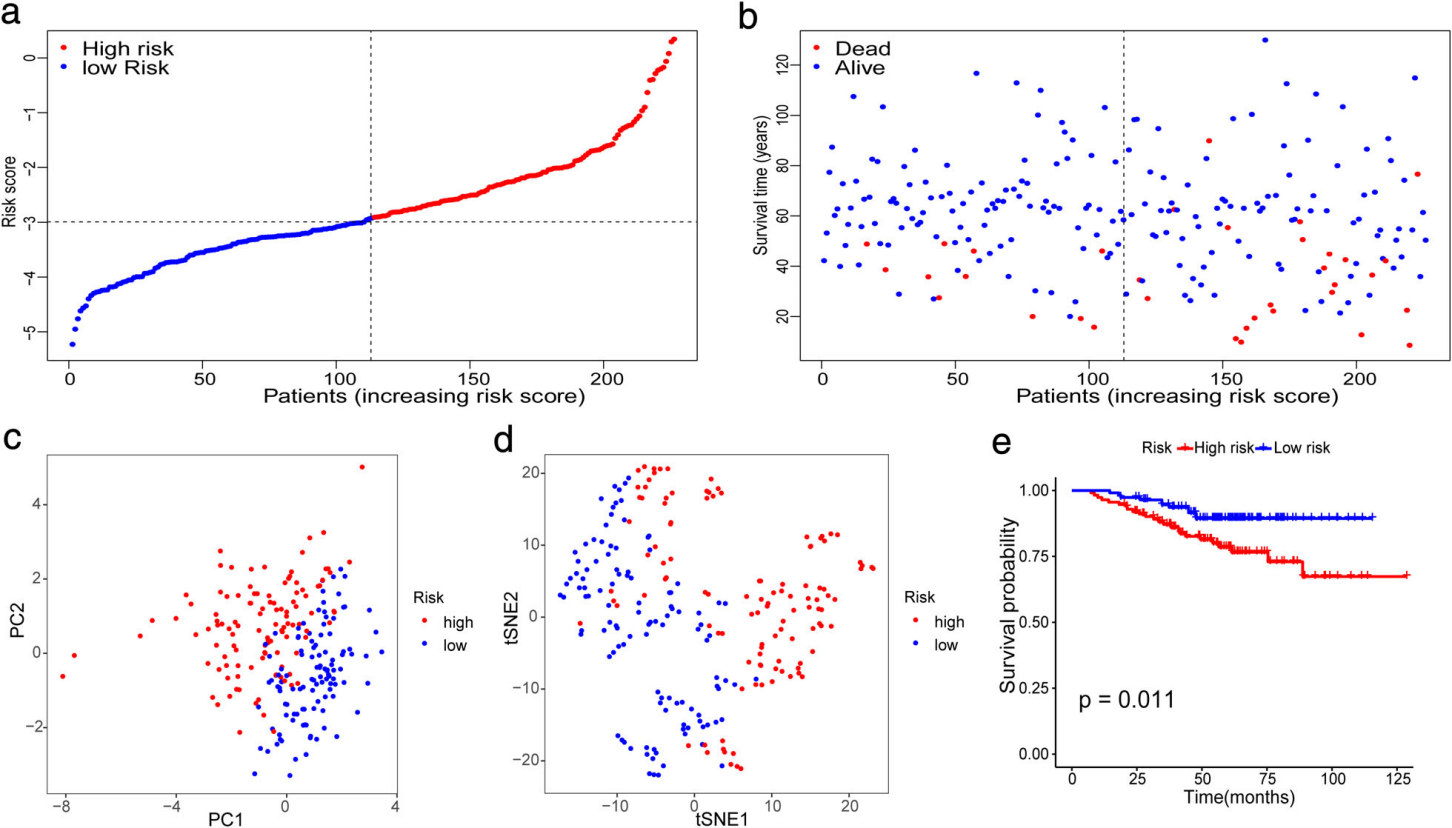
Validation of the 10‐gene signature in the International Cancer Genome Consortium (ICGC) cohort. (a) The distribution and median value of the risk scores in the ICGC cohort. (b) The distributions of overall survival (OS) status, OS and risk score. (c) Principal component analysis (PCA) plot of the ICGC cohort. (d) t‐SNE analysis of the ICGC cohort. (e) Kaplan–Meier curves for the OS of patients in the high‐ and low‐risk groups

To test the robustness of the model constructed from the TCGA cohort, patients from the GEO cohort were also categorized into high‐ or low‐risk groups by the median value calculated with the same formula as that from the TCGA cohort (Figure *5*(a)). Similar to the results obtained from the TCGA cohort, PCA and t‐SNE analysis confirmed that patients in the two subgroups were distributed in discrete directions (Figure *5*(c), *4*(d)). Likewise, patients in the high‐risk group were more likely to encounter death earlier (Figure *5*(b)) and had a reduced survival time compared to those in the low‐risk group (Figure *5*(e), *p* = 0.011).

### Immune‐related functional analyses in the TCGA and GEO cohort

**FIGURE 6:**
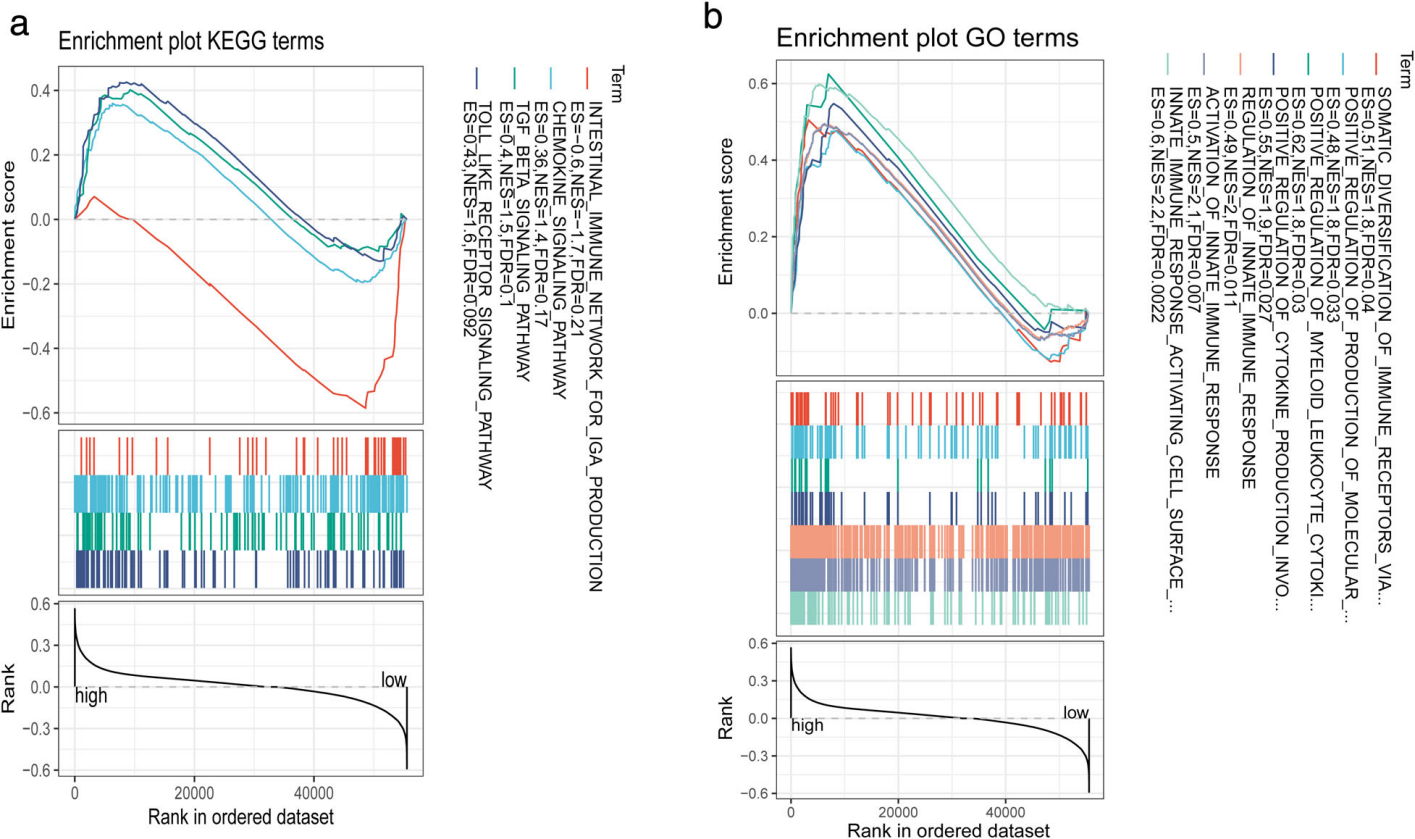
GSEA analyses of KEGG (a) and GO (b) were conducted between the high‐ and low‐risk groups among immune‐related pathway

To elucidate the immune‐related pathways associated with the risk score, the GSEA analyses of GO and KEGG were conducted between the high‐ and low‐risk groups. Interestingly, GSEA results showed that both GO and KEGG had several changes in immune‐related pathways (FDR < 0.25, Figure *6*). Four immune‐related biological processes or molecular functions in KEGG were changed between the high‐ and low‐risk groups in the TCGA cohort, including the intestinal immune network for IGA production, chemokine signaling pathway, TGF beta signaling pathway, and TOLL‐like receptor signaling pathway (FDR < 0.25, Figure *6*(a)). Six immune‐related biological processes or molecular functions in GO were changed between the high‐ and low‐risk groups in the TCGA cohort, including somatic diversification of immune receptors, positive regulation of production of molecular, positive regulation of myeloid leukocyte cytokines, positive regulation of cytokine production, regulation of innate immune response, and activation of the innate immune response (FDR < 0.25, Figure *6*(b)).

**FIGURE 7:**
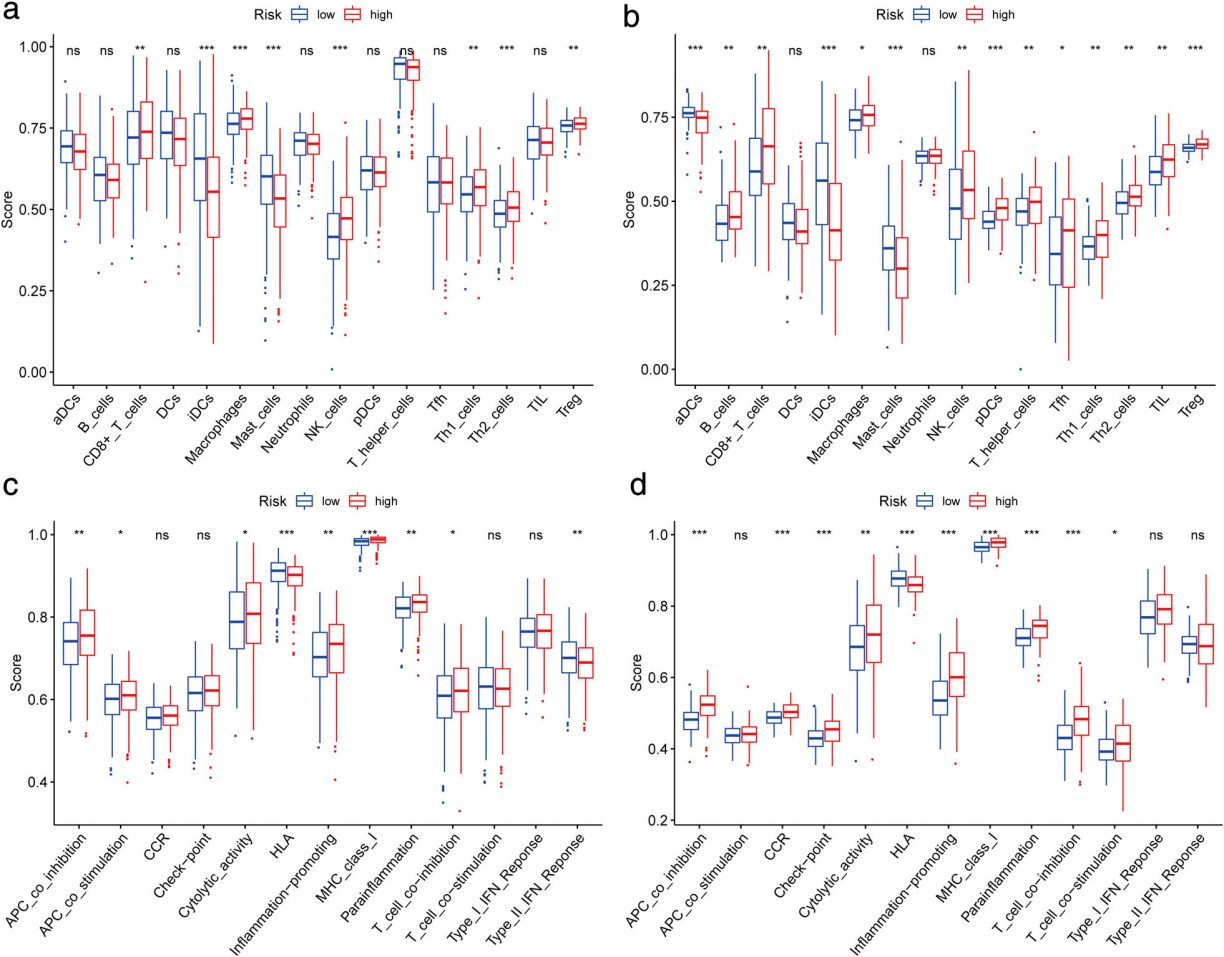
Comparison of the ssGSEA scores between different risk groups in the TCGA cohort (a,c) and GEO cohort (b,d). The scores of 16 immune cells (a,b) and 13 immune‐related functions (c,d) are shown in boxplots. *p*‐values are shown as: Ns, not significant; *p < 0.05; **p < 0.01; ***p < 0.001

To further explore the correlation between the risk score and immune status, we quantified the enrichment scores of diverse immune cell subpopulations, related functions, or pathways with ssGSEA. Interestingly, the score of CD8+ T cells, iDCs, macrophages, mast cells, NK cells, Th1 cells, Th2 cells, Treg, antigen‐presenting cells (APC) coinhibition, cytolytic activity, HLA, inflammation‐promoting, MHC class I, parainflammation, and T cell coinhibition were significantly different between the low‐ and high‐risk groups in both TCGA (all *p* < 0.05, Figure *7*(a), (c)) and GEO cohorts (all *p* < 0.05, Figure *7*(b), (d)). The trend of change in the two cohorts was consistent.

### Combined with clinical and ferroptosis‐related risk scores to construct the prognosis prediction model

**FIGURE 8:**
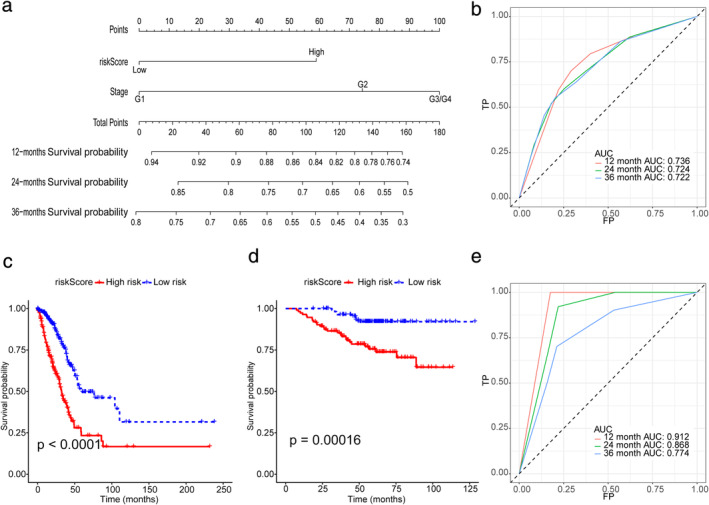
(a) Nomogram for predicting overall survival (OS). (b) ROC curves for predicting OS of the nomogram in the TCGA cohort. (c) Survival curve of OS with the nomogram in the TCGA cohort. The risk score was calculated according to the regression coefficient. The cohort was divided into low‐ and high‐risk score groups by the median risk scores for Kaplan–Meier curve analysis. (d) Survival curve of progression‐free survival (PFS) with the nomogram according to the risk score in the GEO cohort. (e) ROC curves for predicting OS of the nomogram in the GEO cohort

We combined the clinical stage and risk score for multiple Cox regression analysis and constructed a prognosis prediction model. The nomogram was drawn according to the model (Figure *8*(a)). The ROC curve of the model shows that the model has good prognosis prediction ability (12 months ‐ area under curve [AUC]: 0.736, 24 months ‐ AUC: 0.724, 36 months ‐ AUC: 0.722, Figure *8*(b)). According to the median risk score of the new prognostic model, we divided the patients into high‐ and low‐risk groups. Figure *8*(c) shows that the survival of high‐risk patients was lower than that of low‐risk patients. The same result was verified in the GEO cohort (Figure *8*(d)). At the same time, the ROC curve of the geo cohort verified the accuracy of the model in predicting the prognosis (12 months ‐ AUC: 0.912, 24 months ‐ AUC: 0.868, 36 months ‐ AUC: 0.774, Figure *8*(e)).

## DISCUSSION

In the current study, we systematically investigated the expression of 60 FRGs in LUAD tumor tissues and their association with OS. A novel prognostic model integrating 10 FRGs and clinical factors was first constructed and validated in an external cohort. The model has high accuracy in predicting the prognosis of patients with LUAD (AUC: 0.722–0.736, Figure **8**(b)). At the same time, the external verification results of the model also prove that the model has high prediction accuracy (AUC: 0.774–0.912, Figure **8**(e)). The ROC curve results of the external validation set showed that the prediction accuracy of the model in the validation set was higher than that in the training set, which may be because all patients in the validation set were early‐stage patients. This suggests that our model may be more suitable for patients with early‐stage LUAD. In previous studies, the prognosis prediction model was only based on gene signature, completely ignoring the clinical information, and the LASSO Cox regression analysis was not used to select the best gene signature in previous studies.^20^ Our model screened the best gene signature by LASSO Cox regression analysis, calculated its risk score, and divided the patients into groups. A model with more clinical value will then be established by combining this grouping with clinical information.

Functional analyses revealed that immune‐related pathways were enriched for FR‐DEGs signatures. Although a few previous studies^21^, ^22^, ^23^ have indicated that several genes might regulate drug‐induced ferroptosis in LUAD, their value in the survival of patients with LUAD remains largely unknown. To our surprise, most of the FRGs (75%) were differentially expressed between tumor and adjacent nontumorous tissues, and 12 were correlated with OS in the univariate Cox regression analysis. These results significantly indicated the potential role of ferroptosis in LUAD and the possibility of building a prognostic model with these FRGs. The prognostic model proposed in the present study was composed of 10 FRGs (*ALOX15*, *ATP5MC3*, *CISD1*, *DPP4*, *FANCD2*, *GLS2*, *PHKG2*, *ACSL3*, *PEBP1*, *PGD*). These genes can be roughly classified into four categories, including iron metabolism (*FANCD2*, *CISD1*, *PHKG2*), lipid metabolism (*ACSL3*, *PEBP1*), (anti) oxidant metabolism (*ALOX15*), and energy metabolism (*GLS2*, *PGD*, *ATP5MC3*).^8^ These genes were all upregulated in LUAD tumor tissue and were associated with prognosis in the current study. Whether these genes play a role in the prognosis of LUAD patients by influencing the process of ferroptosis remains to be elucidated since few related studies on these genes have been reported.

Although the mechanisms underlying tumor susceptibility to ferroptosis have been an intense area of research in the past few years, the potential modulation between tumor immunity and ferroptosis remains elusive. We performed GSEA analyses and discovered that many immune‐related biological processes and pathways were enriched. This is similar to previous studies.^20^ It is reasonable to assume that ferroptosis may have a close connection with tumor immunity. Interestingly, the contents of the antigen presentation process were significantly different between the low‐risk and high‐risk groups in this study. One possible explanation is that ferroptotic cells release distinct signals, such as lipid mediators, to attract APCs to the site of ferroptotically dying cells.^15^ Previous studies have demonstrated that increased tumor‐associated macrophages^24^, ^25^ or Treg cells^26^ are related to poor prognosis in LUAD patients due to their role in immune invasion. The upregulation of APC coinhibition and T cell coinhibition will decrease immune killing ability and immune escape. Moreover, higher risk scores correlated with lower iDC scores. Therefore, attenuated antitumor immunity in patients at high risk may be an explanation for their poor prognosis. It also provides a potential direction of immunotherapy for LUAD in the future. The clinical model developed in this study can better predict the prognosis of patients with early‐stage lung adenocarcinoma. Nowadays, due to the popularity of next‐generation sequencing, performing expression profiling sequencing of genes in patients can be carried out at a relatively low cost. The model of this study therefore has the potential for widespread generalization, especially in patients with early‐stage lung adenocarcinoma who are able to undergo surgical resection.

However, this study has some limitations. As it was a retrospective study based on public databases, some information, such as specific treatment methods, were unavailable. Moreover, the cohort only included patients with early‐stage LUAD as the validation set, which can only verify that the model has high accuracy in predicting the prognosis of patients with early‐stage LUAD.

In conclusion, the FR‐DEGs signature was related to immune infiltration, and we propose a model based on FR‐DEGs and clinical factors that could be used to predict the prognosis of patients with LUAD.

## CONFLICT OF INTEREST

There are no conflicts of interest.

## Supporting information


**TABLE S1.** Ferrotosis‐related genes.

**TABLE S2.** The annotated gene set for ssGSEA.

## References

[1] Cancer Genome Atlas Research Network . Comprehensive molecular profiling of lung adenocarcinoma. Nature. 2014;511(7511):543–50. 10.1038/nature13385. 25079552 PMC4231481

[2] Sher T , Dy GK , Adjei AA . Small cell lung cancer. Mayo Clin Proc. 2008;83(3):355–67. 10.4065/83.3.355. 18316005

[3] Meza R , Meernik C , Jeon J , Cote ML . Lung cancer incidence trends by gender, race and histology in the United States, 1973‐2010. PLoS One. 2015;10(3):e0121323. 10.1371/journal.pone.0121323. 25822850 PMC4379166

[4] Wakelee HA , Chang ET , Gomez SL , Keegan TH , Feskanich D , Clarke CA , et al. Lung cancer incidence in never smokers. J Clin Oncol. 2007;25(5):472–8. 10.1200/JCO.2006.07.2983. 17290054 PMC2764546

[5] Dixon SJ , Lemberg KM , Lamprecht MR , Skouta R , Zaitsev EM , Gleason CE , et al. Ferroptosis: an iron‐dependent form of nonapoptotic cell death. Cell. 2012;149(5):1060–72. 10.1016/j.cell.2012.03.042. 22632970 PMC3367386

[6] Dixon SJ , Winter GE , Musavi LS , Lee ED , Snijder B , Rebsamen M , et al. Human haploid cell genetics reveals roles for lipid metabolism genes in nonapoptotic cell death. ACS Chem Biol. 2015;10(7):1604–9. 10.1021/acschembio.5b00245. 25965523 PMC4509420

[7] Stockwell BR , Friedmann Angeli JP , Bayir H , Bush AI , Conrad M , Dixon SJ , et al. Ferroptosis: a regulated cell death nexus linking metabolism, redox biology, and disease. Cell. 2017;171(2):273–85. 10.1016/j.cell.2017.09.021. 28985560 PMC5685180

[8] Hassannia B , Vandenabeele P , Vanden BT . Targeting ferroptosis to iron out cancer. Cancer Cell. 2019;35(6):830–49. 10.1016/j.ccell.2019.04.002. 31105042

[9] Liang C , Zhang X , Yang M , Dong X . Recent progress in ferroptosis inducers for cancer therapy. Adv Mater. 2019;31(51):e1904197. 10.1002/adma.201904197. 31595562

[10] Tesfay L , Paul BT , Konstorum A , Deng Z , Cox AO , Lee J , et al. Stearoyl‐CoA desaturase 1 protects ovarian cancer cells from ferroptotic cell death. Cancer Res. 2019;79(20):5355–66. 10.1158/0008-5472.CAN-19-0369. 31270077 PMC6801059

[11] Yang WS , SriRamaratnam R , Welsch ME , Shimada K , Skouta R , Viswanathan VS , et al. Regulation of ferroptotic cancer cell death by GPX4. Cell. 2014;156(1–2):317–31. 10.1016/j.cell.2013.12.010. 24439385 PMC4076414

[12] Yu H , Guo P , Xie X , Wang Y , Chen G . Ferroptosis, a new form of cell death, and its relationships with tumourous diseases. J Cell Mol Med. 2017;21(4):648–57. 10.1111/jcmm.13008. 27860262 PMC5345622

[13] Belavgeni A , Bornstein SR , Linkermann A . Prominin‐2 suppresses ferroptosis sensitivity. Dev Cell. 2019;51(5):548–9. 10.1016/j.devcel.2019.11.004. 31794716

[14] Stockwell BR , Jiang XA . Physiological function for ferroptosis in tumor suppression by the immune system. Cell Metab. 2019;30:14–5. 10.1016/j.cmet.2019.06.012. 31269423 PMC6944065

[15] Friedmann Angeli JP , Krysko DV , Conrad M . Ferroptosis at the crossroads of cancer‐acquired drug resistance and immune evasion. Nat Rev Cancer. 2019;19:405–14. 10.1038/s41568-019-0149-1. 31101865

[16] Yamauchi M , Yamaguchi R , Nakata A , Kohno T , Nagasaki M , Shimamura T , et al. Epidermal growth factor receptor tyrosine kinase defines critical prognostic genes of stage I lung adenocarcinoma. PLoS One. 2012;7(9):e43923. 10.1371/journal.pone.0043923. 23028479 PMC3446964

[17] Simon N , Friedman J , Hastie T , Tibshirani R . Regularization paths for Cox's proportional hazards model via coordinate descent. J Stat Softw. 2011;39(5):1–13. 10.18637/jss.v039.i05. PMC4824408 27065756

[18] Tibshirani R . The lasso method for variable selection in the Cox model. Stat Med. 1997;16(4):385–95. 10.1002/(sici)1097-0258(19970228)16:4<385::aid-sim380>3.0.co;2-3. 9044528

[19] Rooney MS , Shukla SA , Wu CJ , Getz G , Hacohen N . Molecular and genetic properties of tumors associated with local immune cytolytic activity. Cell. 2015;160(1‐2):48–61. 10.1016/j.cell.2014.12.033. 25594174 PMC4856474

[20] Gao X , Tang M , Tian S , Li J , Liu W . A ferroptosis‐related gene signature predicts overall survival in patients with lung adenocarcinoma. Future Oncol. 2021;17(12):1533–44. 10.2217/fon-2020-1113. 33432837

[21] Kuang Y , Wang Q . Iron and lung cancer. Cancer Lett. 2019;464:56–61. 10.1016/j.canlet.2019.08.007. 31437477

[22] Alvarez SW , Sviderskiy VO , Terzi EM , Papagiannakopoulos T , Moreira AL , Adams S , et al. NFS1 undergoes positive selection in lung tumours and protects cells from ferroptosis. Nature. 2017;551(7682):639–43. 10.1038/nature24637. 29168506 PMC5808442

[23] Santarpia M , Aguilar A , Chaib I , Cardona AF , Fancelli S , Laguia F , et al. Non‐small‐cell lung cancer signaling pathways, metabolism, and PD‐1/PD‐L1 antibodies. Cancers. 2020;12(6):1475. 10.3390/cancers12061475. PMC7352732 32516941

[24] Lu CH , Yeh DW , Lai CY , Liu YL , Huang LR , Lee AY , et al. USP17 mediates macrophage‐promoted inflammation and stemness in lung cancer cells by regulating TRAF2/TRAF3 complex formation. Oncogene. 2018;37(49):6327–40. 10.1038/s41388-018-0411-0. 30038267 PMC6283856

[25] Liu X , Wu S , Yang Y , Zhao M , Zhu G , Hou Z . The prognostic landscape of tumor‐infiltrating immune cell and immunomodulators in lung cancer. Biomed Pharmacother. 2017;95:55–61. 10.1016/j.biopha.2017.08.003. 28826097

[26] Tao H , Mimura Y , Aoe K , Kobayashi S , Yamamoto H , Matsuda E , et al. Prognostic potential of FOXP3 expression in non‐small cell lung cancer cells combined with tumor‐infiltrating regulatory T cells. Lung Cancer. 2012;75(1):95–101. 10.1016/j.lungcan.2011.06.002. 21719142

