# Supplementary material for: Prognostic value of ferroptosis‐related genes in patients with lung adenocarcinoma
Source: Thorac Cancer. 2021 May 12;12(12):1890–9. doi: 10.1111/1759-7714.13998 (PMC8201541; doi:10.1111/1759-7714.13998)
Supplement: Supplementary file 2 — TABLE S2. The annotated gene set for ssGSEA. [file TCA-12-1890-s001.docx]

**Table S2:** The annotated gene set for ssGSEA.

| aDCs | APC_co_inhibition | APC_co_stimulation | B_cells | CCR | CD8+_T_cells | Check-point | Cytolytic_activity | DCs | HLA | iDCs | Inflammation-promoting | Macrophages | Mast_cells | MHC_class_I | Neutrophils | NK_cells | Parainflammation | pDCs | T_cell_co-inhibition | T_cell_co-stimulation | T_helper_cells | Tfh | Th1_cells | Th2_cells | TIL | Treg | Type_I_IFN_Reponse | Type_II_IFN_Reponse |
| --- | --- | --- | --- | --- | --- | --- | --- | --- | --- | --- | --- | --- | --- | --- | --- | --- | --- | --- | --- | --- | --- | --- | --- | --- | --- | --- | --- | --- |
| na | na | na | na | na | na | na | na | na | na | na | na | na | na | na | na | na | na | na | na | na | na | na | na | na | na | na | na | na |
| CD83 | C10orf54 | CD40 | BACH2 | CCL16 | CD8A | IDO1 | PRF1 | CCL17 | HLA-E | CD1A | CCL5 | C11orf45 | CMA1 | B2M | EVI2B | KLRC1 | CXCL10 | CLEC4C | BTLA | CD2 | CD4 | PDCD1 | IFNG | PMCH | ITM2C | IL12RB2 | DDX4 | GPR146 |
| LAMP3 | CD274 | CD58 | BANK1 | TPO |  | LAG3 | GZMA | CCL22 | HLA-DPB2 | CD1E | CD19 | CD68 | MS4A2 | HLA-A | HSD17B11 | KLRF1 | PLAT | CXCR3 | C10orf54 | CD226 |  | CXCL13 | TBX21 | LAIR2 | CD38 | TMPRSS6 | IFIT1 | SELP |
| CCL1 | LGALS9 | CD70 | BLK | TGFBR2 |  | CTLA4 |  | CD209 | HLA-C |  | CD8B | CLEC5A | TPSAB1 | TAP1 | KDM6B |  | CCND1 | GZMB | CD160 | CD27 |  | CXCR5 | CTLA4 | SMAD2 | THEMIS2 | CTSC | IFIT2 | AHR |
|  | PDCD1LG2 | ICOSLG | BTLA | CXCL2 |  | TNFRSF9 |  | CCL13 | HLA-J |  | CXCL10 | CYBB |  |  | MEGF9 |  | LGMN | IL3RA | CD244 | CD28 |  |  | STAT4 | CXCR6 | GLYR1 | LAPTM4B | IFIT3 |  |
|  | PVRL3 | SLAMF1 | CD79A | CCL14 |  | ICOS |  |  | HLA-DQB1 |  | CXCL13 | FUCA1 |  |  | MNDA |  | PLAUR | IRF7 | CD274 | CD40LG |  |  | CD38 | GATA3 | ICOS | TFRC | IRF7 |  |
|  |  | TNFSF14 | CD79B | TGFBR3 |  | CD80 |  |  | HLA-DQB2 |  | CXCL9 | GPNMB |  |  | NLRP12 |  | AIM2 | IRF8 | CTLA4 | ICOS |  |  | IL12RB2 | IL26 | F5 | RNF145 | ISG20 |  |
|  |  | TNFSF15 | FCRL1 | IL11RA |  | PDCD1LG2 |  |  | HLA-DQA2 |  | GNLY | HS3ST2 |  |  | PADI4 |  | MMP7 | LILRA4 | HAVCR2 | SLAMF1 |  |  | LTA |  | TIGIT | NETO2 | MX1 |  |
|  |  | TNFSF18 | FCRL3 | CCL11 |  | TIGIT |  |  | HLA-DQA1 |  | GZMB | LGMN |  |  | SELL |  | ICAM1 | PHEX | LAG3 | TNFRSF18 |  |  | CSF2 |  | KLRD1 | ADAT2 | MX2 |  |
|  |  | TNFSF4 | HVCN1 | IL4I1 |  | CD70 |  |  | HLA-A |  | IFNG | MMP9 |  |  | TRANK1 |  | MX2 | PLD4 | LAIR1 | TNFRSF25 |  |  |  |  | IRF4 | CHST2 | RSAD2 |  |
|  |  | TNFSF8 | RALGPS2 | IL33 |  | TNFSF9 |  |  | HLA-DMA |  | IL12A | TM4SF19 |  |  | VNN3 |  | CXCL9 | PTCRA | TIGIT | TNFRSF4 |  |  |  |  | PRKCQ | CTLA4 | TNFSF10 |  |
|  |  | TNFSF9 |  | CXCL12 |  | ICOSLG |  |  | HLA-DOB |  | IL12B |  |  |  |  |  | ANXA1 |  |  | TNFRSF8 |  |  |  |  | FCRL5 | NFE2L3 |  |  |
|  |  |  |  | CXCL10 |  | KIR3DL1 |  |  | HLA-DRB1 |  | IRF1 |  |  |  |  |  | TLR2 |  |  | TNFRSF9 |  |  |  |  | SIRPG | LIMA1 |  |  |
|  |  |  |  | BMPER |  | CD86 |  |  | HLA-H |  | PRF1 |  |  |  |  |  | PLA2G2D |  |  | TNFSF14 |  |  |  |  | LPXN | IL1R2 |  |  |
|  |  |  |  | BMP8A |  | PDCD1 |  |  | HLA-B |  | STAT1 |  |  |  |  |  | ITGA2 |  |  |  |  |  |  |  | IL2RG | ICOS |  |  |
|  |  |  |  | CXCL11 |  | LAIR1 |  |  | HLA-DRB5 |  | TBX21 |  |  |  |  |  | MX1 |  |  |  |  |  |  |  | CCL5 | HSDL2 |  |  |
|  |  |  |  | IL21R |  | TNFRSF8 |  |  | HLA-DOA |  |  |  |  |  |  |  | HMOX1 |  |  |  |  |  |  |  | LCK | HTATIP2 |  |  |
|  |  |  |  | IL17B |  | TNFSF15 |  |  | HLA-DPB1 |  |  |  |  |  |  |  | CD276 |  |  |  |  |  |  |  | TRAF3IP3 | FKBP1A |  |  |
|  |  |  |  | TNFRSF9 |  | TNFRSF14 |  |  | HLA-DRA |  |  |  |  |  |  |  | TIRAP |  |  |  |  |  |  |  | CD86 | TIGIT |  |  |
|  |  |  |  | ILF2 |  | IDO2 |  |  | HLA-DRB6 |  |  |  |  |  |  |  | IL33 |  |  |  |  |  |  |  | MAL | CCR8 |  |  |
|  |  |  |  | CX3CR1 |  | CD276 |  |  | HLA-L |  |  |  |  |  |  |  | PTGES |  |  |  |  |  |  |  | LILRB1 | LTA |  |  |
|  |  |  |  | CCR8 |  | CD40 |  |  | HLA-F |  |  |  |  |  |  |  | TNFRSF12A |  |  |  |  |  |  |  | DOK2 | SLC35F2 |  |  |
|  |  |  |  | TNFSF12 |  | TNFRSF4 |  |  | HLA-G |  |  |  |  |  |  |  | SCARB1 |  |  |  |  |  |  |  | CD6 | IL21R |  |  |
|  |  |  |  | CSF3 |  | TNFSF14 |  |  | HLA-DMB |  |  |  |  |  |  |  | CD14 |  |  |  |  |  |  |  | PAG1 | AHCYL1 |  |  |
|  |  |  |  | TNFSF4 |  | HHLA2 |  |  | HLA-DPA1 |  |  |  |  |  |  |  | BLNK |  |  |  |  |  |  |  | LAX1 | SOCS2 |  |  |
|  |  |  |  | BMP3 |  | CD244 |  |  |  |  |  |  |  |  |  |  | IFIT3 |  |  |  |  |  |  |  | PLEK | ETV7 |  |  |
|  |  |  |  | CX3CL1 |  | CD274 |  |  |  |  |  |  |  |  |  |  | RETNLB |  |  |  |  |  |  |  | PIK3CD | BCL2L1 |  |  |
|  |  |  |  | BMP5 |  | HAVCR2 |  |  |  |  |  |  |  |  |  |  | IFIT2 |  |  |  |  |  |  |  | SLAMF1 | RRAGB |  |  |
|  |  |  |  | CXCR2 |  | CD27 |  |  |  |  |  |  |  |  |  |  | ISG15 |  |  |  |  |  |  |  | XCL1 | ACSL4 |  |  |
|  |  |  |  | TNFRSF10D |  | BTLA |  |  |  |  |  |  |  |  |  |  | OAS2 |  |  |  |  |  |  |  | GPR171 | CHRNA6 |  |  |
|  |  |  |  | BMP2 |  | LGALS9 |  |  |  |  |  |  |  |  |  |  | REL |  |  |  |  |  |  |  | XCL2 | BATF |  |  |
|  |  |  |  | CXCL14 |  | TMIGD2 |  |  |  |  |  |  |  |  |  |  | OAS3 |  |  |  |  |  |  |  | TBX21 | LAX1 |  |  |
|  |  |  |  | CCL28 |  | CD28 |  |  |  |  |  |  |  |  |  |  | CD44 |  |  |  |  |  |  |  | CD2 | ADPRH |  |  |
|  |  |  |  | CXCL3 |  | CD48 |  |  |  |  |  |  |  |  |  |  | PPARG |  |  |  |  |  |  |  | CD53 | TNFRSF4 |  |  |
|  |  |  |  | BMP6 |  | TNFRSF25 |  |  |  |  |  |  |  |  |  |  | BST2 |  |  |  |  |  |  |  | KLHL6 | ANKRD10 |  |  |
|  |  |  |  | CCL21 |  | CD40LG |  |  |  |  |  |  |  |  |  |  | OAS1 |  |  |  |  |  |  |  | SLAMF6 | CD274 |  |  |
|  |  |  |  | CXCL9 |  | ADORA2A |  |  |  |  |  |  |  |  |  |  | NOX1 |  |  |  |  |  |  |  | CD40 | CASP1 |  |  |
|  |  |  |  | CCL23 |  | VTCN1 |  |  |  |  |  |  |  |  |  |  | PLA2G2A |  |  |  |  |  |  |  | SIT1 | LY75 |  |  |
|  |  |  |  | IL6 |  | CD160 |  |  |  |  |  |  |  |  |  |  | IFIT1 |  |  |  |  |  |  |  | TNFRSF4 | NPTN |  |  |
|  |  |  |  | TNFRSF18 |  | CD44 |  |  |  |  |  |  |  |  |  |  | IFITM3 |  |  |  |  |  |  |  | CD79A | SSTR3 |  |  |
|  |  |  |  | IL17RD |  | TNFSF18 |  |  |  |  |  |  |  |  |  |  | IL1RN |  |  |  |  |  |  |  | CD247 | GRSF1 |  |  |
|  |  |  |  | IL17D |  | TNFRSF18 |  |  |  |  |  |  |  |  |  |  |  |  |  |  |  |  |  |  | LCP2 | CSF2RB |  |  |
|  |  |  |  | IL27 |  | BTNL2 |  |  |  |  |  |  |  |  |  |  |  |  |  |  |  |  |  |  | CD3D | TMEM184C |  |  |
|  |  |  |  | CCL7 |  | C10orf54 |  |  |  |  |  |  |  |  |  |  |  |  |  |  |  |  |  |  | CD27 | NDFIP2 |  |  |
|  |  |  |  | IL1R1 |  | CD200R1 |  |  |  |  |  |  |  |  |  |  |  |  |  |  |  |  |  |  | SH2D1A | ZBTB38 |  |  |
|  |  |  |  | CXCR4 |  | TNFSF4 |  |  |  |  |  |  |  |  |  |  |  |  |  |  |  |  |  |  | FYB | ERI1 |  |  |
|  |  |  |  | CXCR2P1 |  | CD200 |  |  |  |  |  |  |  |  |  |  |  |  |  |  |  |  |  |  | ARHGAP30 | TRAF3 |  |  |
|  |  |  |  | TGFB1I1 |  | NRP1 |  |  |  |  |  |  |  |  |  |  |  |  |  |  |  |  |  |  | ACAP1 | NAB1 |  |  |
|  |  |  |  | IFNGR1 |  |  |  |  |  |  |  |  |  |  |  |  |  |  |  |  |  |  |  |  | CST7 | HS3ST3B1 |  |  |
|  |  |  |  | IL9R |  |  |  |  |  |  |  |  |  |  |  |  |  |  |  |  |  |  |  |  | CD3G | LAYN |  |  |
|  |  |  |  | IL1RAPL1 |  |  |  |  |  |  |  |  |  |  |  |  |  |  |  |  |  |  |  |  | IL2RB | JAK1 |  |  |
|  |  |  |  | IL11 |  |  |  |  |  |  |  |  |  |  |  |  |  |  |  |  |  |  |  |  | CD3E | VDR |  |  |
|  |  |  |  | CSF1 |  |  |  |  |  |  |  |  |  |  |  |  |  |  |  |  |  |  |  |  | FCRL3 | LEPROT |  |  |
|  |  |  |  | IL20RA |  |  |  |  |  |  |  |  |  |  |  |  |  |  |  |  |  |  |  |  | CORO1A | GCNT1 |  |  |
|  |  |  |  | IL25 |  |  |  |  |  |  |  |  |  |  |  |  |  |  |  |  |  |  |  |  | ITK | PTPRJ |  |  |
|  |  |  |  | TNFRSF4 |  |  |  |  |  |  |  |  |  |  |  |  |  |  |  |  |  |  |  |  | TCL1A | IKZF2 |  |  |
|  |  |  |  | IL18 |  |  |  |  |  |  |  |  |  |  |  |  |  |  |  |  |  |  |  |  | CYBB | CSF1 |  |  |
|  |  |  |  | ILF3 |  |  |  |  |  |  |  |  |  |  |  |  |  |  |  |  |  |  |  |  | CSF2RB | ENTPD1 |  |  |
|  |  |  |  | CCL20 |  |  |  |  |  |  |  |  |  |  |  |  |  |  |  |  |  |  |  |  | IKZF1 | TNFRSF18 |  |  |
|  |  |  |  | TNFRSF12A |  |  |  |  |  |  |  |  |  |  |  |  |  |  |  |  |  |  |  |  | NCF4 | METTL7A |  |  |
|  |  |  |  | IL6ST |  |  |  |  |  |  |  |  |  |  |  |  |  |  |  |  |  |  |  |  | DOCK2 | KSR1 |  |  |
|  |  |  |  | CXCL13 |  |  |  |  |  |  |  |  |  |  |  |  |  |  |  |  |  |  |  |  | CCR2 | SSH1 |  |  |
|  |  |  |  | IL12B |  |  |  |  |  |  |  |  |  |  |  |  |  |  |  |  |  |  |  |  | PTPRC | CADM1 |  |  |
|  |  |  |  | TNFRSF8 |  |  |  |  |  |  |  |  |  |  |  |  |  |  |  |  |  |  |  |  | PLAC8 | IL1R1 |  |  |
|  |  |  |  | IL6R |  |  |  |  |  |  |  |  |  |  |  |  |  |  |  |  |  |  |  |  | NCKAP1L | ACP5 |  |  |
|  |  |  |  | BMPR2 |  |  |  |  |  |  |  |  |  |  |  |  |  |  |  |  |  |  |  |  | IL7R | CHST7 |  |  |
|  |  |  |  | IFNE |  |  |  |  |  |  |  |  |  |  |  |  |  |  |  |  |  |  |  |  | 6-Sep | THADA |  |  |
|  |  |  |  | IL1RAPL2 |  |  |  |  |  |  |  |  |  |  |  |  |  |  |  |  |  |  |  |  | CD28 | CD177 |  |  |
|  |  |  |  | IL3RA |  |  |  |  |  |  |  |  |  |  |  |  |  |  |  |  |  |  |  |  | STAT4 | NFAT5 |  |  |
|  |  |  |  | BMP4 |  |  |  |  |  |  |  |  |  |  |  |  |  |  |  |  |  |  |  |  | CD8A | ZNF282 |  |  |
|  |  |  |  | CCL24 |  |  |  |  |  |  |  |  |  |  |  |  |  |  |  |  |  |  |  |  | LY9 | MAGEH1 |  |  |
|  |  |  |  | TNFSF13B |  |  |  |  |  |  |  |  |  |  |  |  |  |  |  |  |  |  |  |  | CD48 |  |  |  |
|  |  |  |  | CCR4 |  |  |  |  |  |  |  |  |  |  |  |  |  |  |  |  |  |  |  |  | HCST |  |  |  |
|  |  |  |  | IL2RA |  |  |  |  |  |  |  |  |  |  |  |  |  |  |  |  |  |  |  |  | PTPRCAP |  |  |  |
|  |  |  |  | IL32 |  |  |  |  |  |  |  |  |  |  |  |  |  |  |  |  |  |  |  |  | SASH3 |  |  |  |
|  |  |  |  | TNFRSF10C |  |  |  |  |  |  |  |  |  |  |  |  |  |  |  |  |  |  |  |  | ARHGAP25 |  |  |  |
|  |  |  |  | IL22RA1 |  |  |  |  |  |  |  |  |  |  |  |  |  |  |  |  |  |  |  |  | LAT |  |  |  |
|  |  |  |  | BMPR1A |  |  |  |  |  |  |  |  |  |  |  |  |  |  |  |  |  |  |  |  | TRAT1 |  |  |  |
|  |  |  |  | CXCR5 |  |  |  |  |  |  |  |  |  |  |  |  |  |  |  |  |  |  |  |  | IL10RA |  |  |  |
|  |  |  |  | CXCR3 |  |  |  |  |  |  |  |  |  |  |  |  |  |  |  |  |  |  |  |  | PAX5 |  |  |  |
|  |  |  |  | IFNA8 |  |  |  |  |  |  |  |  |  |  |  |  |  |  |  |  |  |  |  |  | CCR7 |  |  |  |
|  |  |  |  | IL17REL |  |  |  |  |  |  |  |  |  |  |  |  |  |  |  |  |  |  |  |  | DOCK11 |  |  |  |
|  |  |  |  | IFNB1 |  |  |  |  |  |  |  |  |  |  |  |  |  |  |  |  |  |  |  |  | PARVG |  |  |  |
|  |  |  |  | IFNAR1 |  |  |  |  |  |  |  |  |  |  |  |  |  |  |  |  |  |  |  |  | SPNS1 |  |  |  |
|  |  |  |  | TNFRSF1B |  |  |  |  |  |  |  |  |  |  |  |  |  |  |  |  |  |  |  |  | CD52 |  |  |  |
|  |  |  |  | CCL17 |  |  |  |  |  |  |  |  |  |  |  |  |  |  |  |  |  |  |  |  | HCLS1 |  |  |  |
|  |  |  |  | IFNL1 |  |  |  |  |  |  |  |  |  |  |  |  |  |  |  |  |  |  |  |  | ARHGAP9 |  |  |  |
|  |  |  |  | IL16 |  |  |  |  |  |  |  |  |  |  |  |  |  |  |  |  |  |  |  |  | GIMAP6 |  |  |  |
|  |  |  |  | IL1RL1 |  |  |  |  |  |  |  |  |  |  |  |  |  |  |  |  |  |  |  |  | PRKCB |  |  |  |
|  |  |  |  | ILK |  |  |  |  |  |  |  |  |  |  |  |  |  |  |  |  |  |  |  |  | MS4A1 |  |  |  |
|  |  |  |  | CCL25 |  |  |  |  |  |  |  |  |  |  |  |  |  |  |  |  |  |  |  |  | GPR18 |  |  |  |
|  |  |  |  | ILDR2 |  |  |  |  |  |  |  |  |  |  |  |  |  |  |  |  |  |  |  |  | TBC1D10C |  |  |  |
|  |  |  |  | CXCR1 |  |  |  |  |  |  |  |  |  |  |  |  |  |  |  |  |  |  |  |  | GVINP1 |  |  |  |
|  |  |  |  | IL36RN |  |  |  |  |  |  |  |  |  |  |  |  |  |  |  |  |  |  |  |  | P2RY8 |  |  |  |
|  |  |  |  | IL34 |  |  |  |  |  |  |  |  |  |  |  |  |  |  |  |  |  |  |  |  | EVI2B |  |  |  |
|  |  |  |  | TGFB1 |  |  |  |  |  |  |  |  |  |  |  |  |  |  |  |  |  |  |  |  | VAMP5 |  |  |  |
|  |  |  |  | IFNG |  |  |  |  |  |  |  |  |  |  |  |  |  |  |  |  |  |  |  |  | KLRK1 |  |  |  |
|  |  |  |  | IL19 |  |  |  |  |  |  |  |  |  |  |  |  |  |  |  |  |  |  |  |  | SELL |  |  |  |
|  |  |  |  | ILKAP |  |  |  |  |  |  |  |  |  |  |  |  |  |  |  |  |  |  |  |  | MPEG1 |  |  |  |
|  |  |  |  | BMP2K |  |  |  |  |  |  |  |  |  |  |  |  |  |  |  |  |  |  |  |  | MS4A6A |  |  |  |
|  |  |  |  | CCR10 |  |  |  |  |  |  |  |  |  |  |  |  |  |  |  |  |  |  |  |  | ARHGAP15 |  |  |  |
|  |  |  |  | ILDR1 |  |  |  |  |  |  |  |  |  |  |  |  |  |  |  |  |  |  |  |  | MFNG |  |  |  |
|  |  |  |  | EPO |  |  |  |  |  |  |  |  |  |  |  |  |  |  |  |  |  |  |  |  | GZMK |  |  |  |
|  |  |  |  | CCR7 |  |  |  |  |  |  |  |  |  |  |  |  |  |  |  |  |  |  |  |  | SELPLG |  |  |  |
|  |  |  |  | IL17C |  |  |  |  |  |  |  |  |  |  |  |  |  |  |  |  |  |  |  |  | TARP |  |  |  |
|  |  |  |  | IL23A |  |  |  |  |  |  |  |  |  |  |  |  |  |  |  |  |  |  |  |  | GIMAP7 |  |  |  |
|  |  |  |  | CCR5 |  |  |  |  |  |  |  |  |  |  |  |  |  |  |  |  |  |  |  |  | FAM65B |  |  |  |
|  |  |  |  | IL7 |  |  |  |  |  |  |  |  |  |  |  |  |  |  |  |  |  |  |  |  | INPP5D |  |  |  |
|  |  |  |  | EPOR |  |  |  |  |  |  |  |  |  |  |  |  |  |  |  |  |  |  |  |  | ITGA4 |  |  |  |
|  |  |  |  | CCL13 |  |  |  |  |  |  |  |  |  |  |  |  |  |  |  |  |  |  |  |  | MZB1 |  |  |  |
|  |  |  |  | IL2RG |  |  |  |  |  |  |  |  |  |  |  |  |  |  |  |  |  |  |  |  | GPSM3 |  |  |  |
|  |  |  |  | IL31RA |  |  |  |  |  |  |  |  |  |  |  |  |  |  |  |  |  |  |  |  | STK10 |  |  |  |
|  |  |  |  | TNFAIP6 |  |  |  |  |  |  |  |  |  |  |  |  |  |  |  |  |  |  |  |  | CLEC2D |  |  |  |
|  |  |  |  | IFNL2 |  |  |  |  |  |  |  |  |  |  |  |  |  |  |  |  |  |  |  |  | IL16 |  |  |  |
|  |  |  |  | BMP1 |  |  |  |  |  |  |  |  |  |  |  |  |  |  |  |  |  |  |  |  | NLRC3 |  |  |  |
|  |  |  |  | IL12RB1 |  |  |  |  |  |  |  |  |  |  |  |  |  |  |  |  |  |  |  |  | GIMAP5 |  |  |  |
|  |  |  |  | TNFAIP8 |  |  |  |  |  |  |  |  |  |  |  |  |  |  |  |  |  |  |  |  | GIMAP4 |  |  |  |
|  |  |  |  | IL4R |  |  |  |  |  |  |  |  |  |  |  |  |  |  |  |  |  |  |  |  | IFFO1 |  |  |  |
|  |  |  |  | TNFRSF6B |  |  |  |  |  |  |  |  |  |  |  |  |  |  |  |  |  |  |  |  | CFH |  |  |  |
|  |  |  |  | TNFAIP8L1 |  |  |  |  |  |  |  |  |  |  |  |  |  |  |  |  |  |  |  |  | PVRIG |  |  |  |
|  |  |  |  | TNFRSF10B |  |  |  |  |  |  |  |  |  |  |  |  |  |  |  |  |  |  |  |  | CFHR1 |  |  |  |
|  |  |  |  | IFNL3 |  |  |  |  |  |  |  |  |  |  |  |  |  |  |  |  |  |  |  |  |  |  |  |  |
|  |  |  |  | CCL5 |  |  |  |  |  |  |  |  |  |  |  |  |  |  |  |  |  |  |  |  |  |  |  |  |
|  |  |  |  | CXCL6 |  |  |  |  |  |  |  |  |  |  |  |  |  |  |  |  |  |  |  |  |  |  |  |  |
|  |  |  |  | CXCL1 |  |  |  |  |  |  |  |  |  |  |  |  |  |  |  |  |  |  |  |  |  |  |  |  |
|  |  |  |  | CCR3 |  |  |  |  |  |  |  |  |  |  |  |  |  |  |  |  |  |  |  |  |  |  |  |  |
|  |  |  |  | TNFSF11 |  |  |  |  |  |  |  |  |  |  |  |  |  |  |  |  |  |  |  |  |  |  |  |  |
|  |  |  |  | CSF1R |  |  |  |  |  |  |  |  |  |  |  |  |  |  |  |  |  |  |  |  |  |  |  |  |
|  |  |  |  | IL21 |  |  |  |  |  |  |  |  |  |  |  |  |  |  |  |  |  |  |  |  |  |  |  |  |
|  |  |  |  | IL1RAP |  |  |  |  |  |  |  |  |  |  |  |  |  |  |  |  |  |  |  |  |  |  |  |  |
|  |  |  |  | IL12RB2 |  |  |  |  |  |  |  |  |  |  |  |  |  |  |  |  |  |  |  |  |  |  |  |  |
|  |  |  |  | CCL1 |  |  |  |  |  |  |  |  |  |  |  |  |  |  |  |  |  |  |  |  |  |  |  |  |
|  |  |  |  | IL17RA |  |  |  |  |  |  |  |  |  |  |  |  |  |  |  |  |  |  |  |  |  |  |  |  |
|  |  |  |  | CCR1 |  |  |  |  |  |  |  |  |  |  |  |  |  |  |  |  |  |  |  |  |  |  |  |  |
|  |  |  |  | IL1RN |  |  |  |  |  |  |  |  |  |  |  |  |  |  |  |  |  |  |  |  |  |  |  |  |
|  |  |  |  | TNFRSF11B |  |  |  |  |  |  |  |  |  |  |  |  |  |  |  |  |  |  |  |  |  |  |  |  |
|  |  |  |  | TNFRSF14 |  |  |  |  |  |  |  |  |  |  |  |  |  |  |  |  |  |  |  |  |  |  |  |  |
|  |  |  |  | IL13 |  |  |  |  |  |  |  |  |  |  |  |  |  |  |  |  |  |  |  |  |  |  |  |  |
|  |  |  |  | IL2RB |  |  |  |  |  |  |  |  |  |  |  |  |  |  |  |  |  |  |  |  |  |  |  |  |
|  |  |  |  | BMP8B |  |  |  |  |  |  |  |  |  |  |  |  |  |  |  |  |  |  |  |  |  |  |  |  |
|  |  |  |  | CCL2 |  |  |  |  |  |  |  |  |  |  |  |  |  |  |  |  |  |  |  |  |  |  |  |  |
|  |  |  |  | IL24 |  |  |  |  |  |  |  |  |  |  |  |  |  |  |  |  |  |  |  |  |  |  |  |  |
|  |  |  |  | IL18RAP |  |  |  |  |  |  |  |  |  |  |  |  |  |  |  |  |  |  |  |  |  |  |  |  |
|  |  |  |  | TGFBI |  |  |  |  |  |  |  |  |  |  |  |  |  |  |  |  |  |  |  |  |  |  |  |  |
|  |  |  |  | TNFSF10 |  |  |  |  |  |  |  |  |  |  |  |  |  |  |  |  |  |  |  |  |  |  |  |  |
|  |  |  |  | TNFRSF11A |  |  |  |  |  |  |  |  |  |  |  |  |  |  |  |  |  |  |  |  |  |  |  |  |
|  |  |  |  | CXCL5 |  |  |  |  |  |  |  |  |  |  |  |  |  |  |  |  |  |  |  |  |  |  |  |  |
|  |  |  |  | IL5RA |  |  |  |  |  |  |  |  |  |  |  |  |  |  |  |  |  |  |  |  |  |  |  |  |
|  |  |  |  | TNFSF9 |  |  |  |  |  |  |  |  |  |  |  |  |  |  |  |  |  |  |  |  |  |  |  |  |
|  |  |  |  | IL1RL2 |  |  |  |  |  |  |  |  |  |  |  |  |  |  |  |  |  |  |  |  |  |  |  |  |
|  |  |  |  | TNFRSF13C |  |  |  |  |  |  |  |  |  |  |  |  |  |  |  |  |  |  |  |  |  |  |  |  |
|  |  |  |  | IL36G |  |  |  |  |  |  |  |  |  |  |  |  |  |  |  |  |  |  |  |  |  |  |  |  |
|  |  |  |  | IL15RA |  |  |  |  |  |  |  |  |  |  |  |  |  |  |  |  |  |  |  |  |  |  |  |  |
|  |  |  |  | TNFRSF21 |  |  |  |  |  |  |  |  |  |  |  |  |  |  |  |  |  |  |  |  |  |  |  |  |
|  |  |  |  | CXCL8 |  |  |  |  |  |  |  |  |  |  |  |  |  |  |  |  |  |  |  |  |  |  |  |  |
|  |  |  |  | IL22RA2 |  |  |  |  |  |  |  |  |  |  |  |  |  |  |  |  |  |  |  |  |  |  |  |  |
|  |  |  |  | TNFAIP8L2 |  |  |  |  |  |  |  |  |  |  |  |  |  |  |  |  |  |  |  |  |  |  |  |  |
|  |  |  |  | IL18R1 |  |  |  |  |  |  |  |  |  |  |  |  |  |  |  |  |  |  |  |  |  |  |  |  |
|  |  |  |  | IFNLR1 |  |  |  |  |  |  |  |  |  |  |  |  |  |  |  |  |  |  |  |  |  |  |  |  |
|  |  |  |  | CXCR6 |  |  |  |  |  |  |  |  |  |  |  |  |  |  |  |  |  |  |  |  |  |  |  |  |
|  |  |  |  | CCL3L3 |  |  |  |  |  |  |  |  |  |  |  |  |  |  |  |  |  |  |  |  |  |  |  |  |
|  |  |  |  | TNFRSF1A |  |  |  |  |  |  |  |  |  |  |  |  |  |  |  |  |  |  |  |  |  |  |  |  |
|  |  |  |  | IL17RE |  |  |  |  |  |  |  |  |  |  |  |  |  |  |  |  |  |  |  |  |  |  |  |  |
|  |  |  |  | IFNGR2 |  |  |  |  |  |  |  |  |  |  |  |  |  |  |  |  |  |  |  |  |  |  |  |  |
|  |  |  |  | IL17RC |  |  |  |  |  |  |  |  |  |  |  |  |  |  |  |  |  |  |  |  |  |  |  |  |
|  |  |  |  | TNFAIP8L3 |  |  |  |  |  |  |  |  |  |  |  |  |  |  |  |  |  |  |  |  |  |  |  |  |
|  |  |  |  | ILVBL |  |  |  |  |  |  |  |  |  |  |  |  |  |  |  |  |  |  |  |  |  |  |  |  |
|  |  |  |  | TGFBRAP1 |  |  |  |  |  |  |  |  |  |  |  |  |  |  |  |  |  |  |  |  |  |  |  |  |
|  |  |  |  | CCL4L1 |  |  |  |  |  |  |  |  |  |  |  |  |  |  |  |  |  |  |  |  |  |  |  |  |
|  |  |  |  | CSF2RA |  |  |  |  |  |  |  |  |  |  |  |  |  |  |  |  |  |  |  |  |  |  |  |  |
|  |  |  |  | CCRN4L |  |  |  |  |  |  |  |  |  |  |  |  |  |  |  |  |  |  |  |  |  |  |  |  |
|  |  |  |  | CCL26 |  |  |  |  |  |  |  |  |  |  |  |  |  |  |  |  |  |  |  |  |  |  |  |  |
|  |  |  |  | TNFAIP1 |  |  |  |  |  |  |  |  |  |  |  |  |  |  |  |  |  |  |  |  |  |  |  |  |
|  |  |  |  | CCRL2 |  |  |  |  |  |  |  |  |  |  |  |  |  |  |  |  |  |  |  |  |  |  |  |  |
|  |  |  |  | IFNA10 |  |  |  |  |  |  |  |  |  |  |  |  |  |  |  |  |  |  |  |  |  |  |  |  |
|  |  |  |  | TNFRSF17 |  |  |  |  |  |  |  |  |  |  |  |  |  |  |  |  |  |  |  |  |  |  |  |  |
|  |  |  |  | IFNA13 |  |  |  |  |  |  |  |  |  |  |  |  |  |  |  |  |  |  |  |  |  |  |  |  |
|  |  |  |  | IL20 |  |  |  |  |  |  |  |  |  |  |  |  |  |  |  |  |  |  |  |  |  |  |  |  |
|  |  |  |  | IL18BP |  |  |  |  |  |  |  |  |  |  |  |  |  |  |  |  |  |  |  |  |  |  |  |  |
|  |  |  |  | CCL3L1 |  |  |  |  |  |  |  |  |  |  |  |  |  |  |  |  |  |  |  |  |  |  |  |  |
|  |  |  |  | TNFSF12-TNFSF13 | |  |  |  |  |  |  |  |  |  |  |  |  |  |  |  |  |  |  |  |  |  |  |  |
|  |  |  |  | IL5 |  |  |  |  |  |  |  |  |  |  |  |  |  |  |  |  |  |  |  |  |  |  |  |  |
|  |  |  |  | IL23R |  |  |  |  |  |  |  |  |  |  |  |  |  |  |  |  |  |  |  |  |  |  |  |  |
|  |  |  |  | IL26 |  |  |  |  |  |  |  |  |  |  |  |  |  |  |  |  |  |  |  |  |  |  |  |  |
|  |  |  |  | TNF |  |  |  |  |  |  |  |  |  |  |  |  |  |  |  |  |  |  |  |  |  |  |  |  |
|  |  |  |  | TGFA |  |  |  |  |  |  |  |  |  |  |  |  |  |  |  |  |  |  |  |  |  |  |  |  |
|  |  |  |  | CSF2 |  |  |  |  |  |  |  |  |  |  |  |  |  |  |  |  |  |  |  |  |  |  |  |  |
|  |  |  |  | IL1F10 |  |  |  |  |  |  |  |  |  |  |  |  |  |  |  |  |  |  |  |  |  |  |  |  |
|  |  |  |  | CXCL17 |  |  |  |  |  |  |  |  |  |  |  |  |  |  |  |  |  |  |  |  |  |  |  |  |
|  |  |  |  | TNFSF13 |  |  |  |  |  |  |  |  |  |  |  |  |  |  |  |  |  |  |  |  |  |  |  |  |
|  |  |  |  | IFNA4 |  |  |  |  |  |  |  |  |  |  |  |  |  |  |  |  |  |  |  |  |  |  |  |  |
|  |  |  |  | IL37 |  |  |  |  |  |  |  |  |  |  |  |  |  |  |  |  |  |  |  |  |  |  |  |  |
|  |  |  |  | IL12A |  |  |  |  |  |  |  |  |  |  |  |  |  |  |  |  |  |  |  |  |  |  |  |  |
|  |  |  |  | IL7R |  |  |  |  |  |  |  |  |  |  |  |  |  |  |  |  |  |  |  |  |  |  |  |  |
|  |  |  |  | IFNA1 |  |  |  |  |  |  |  |  |  |  |  |  |  |  |  |  |  |  |  |  |  |  |  |  |
|  |  |  |  | IL1A |  |  |  |  |  |  |  |  |  |  |  |  |  |  |  |  |  |  |  |  |  |  |  |  |
|  |  |  |  | IL4 |  |  |  |  |  |  |  |  |  |  |  |  |  |  |  |  |  |  |  |  |  |  |  |  |
|  |  |  |  | IL2 |  |  |  |  |  |  |  |  |  |  |  |  |  |  |  |  |  |  |  |  |  |  |  |  |
|  |  |  |  | CCL22 |  |  |  |  |  |  |  |  |  |  |  |  |  |  |  |  |  |  |  |  |  |  |  |  |
|  |  |  |  | CSF3R |  |  |  |  |  |  |  |  |  |  |  |  |  |  |  |  |  |  |  |  |  |  |  |  |
|  |  |  |  | IL10 |  |  |  |  |  |  |  |  |  |  |  |  |  |  |  |  |  |  |  |  |  |  |  |  |
|  |  |  |  | IFNK |  |  |  |  |  |  |  |  |  |  |  |  |  |  |  |  |  |  |  |  |  |  |  |  |
|  |  |  |  | TGFB2 |  |  |  |  |  |  |  |  |  |  |  |  |  |  |  |  |  |  |  |  |  |  |  |  |
|  |  |  |  | IL1R2 |  |  |  |  |  |  |  |  |  |  |  |  |  |  |  |  |  |  |  |  |  |  |  |  |
|  |  |  |  | IL1B |  |  |  |  |  |  |  |  |  |  |  |  |  |  |  |  |  |  |  |  |  |  |  |  |
|  |  |  |  | IL17F |  |  |  |  |  |  |  |  |  |  |  |  |  |  |  |  |  |  |  |  |  |  |  |  |
|  |  |  |  | IL27RA |  |  |  |  |  |  |  |  |  |  |  |  |  |  |  |  |  |  |  |  |  |  |  |  |
|  |  |  |  | IL15 |  |  |  |  |  |  |  |  |  |  |  |  |  |  |  |  |  |  |  |  |  |  |  |  |
|  |  |  |  | TNFSF8 |  |  |  |  |  |  |  |  |  |  |  |  |  |  |  |  |  |  |  |  |  |  |  |  |
|  |  |  |  | IL36B |  |  |  |  |  |  |  |  |  |  |  |  |  |  |  |  |  |  |  |  |  |  |  |  |
|  |  |  |  | XCL1 |  |  |  |  |  |  |  |  |  |  |  |  |  |  |  |  |  |  |  |  |  |  |  |  |
|  |  |  |  | CXCL16 |  |  |  |  |  |  |  |  |  |  |  |  |  |  |  |  |  |  |  |  |  |  |  |  |
|  |  |  |  | TNFRSF19 |  |  |  |  |  |  |  |  |  |  |  |  |  |  |  |  |  |  |  |  |  |  |  |  |
|  |  |  |  | IL3 |  |  |  |  |  |  |  |  |  |  |  |  |  |  |  |  |  |  |  |  |  |  |  |  |
|  |  |  |  | CCL3 |  |  |  |  |  |  |  |  |  |  |  |  |  |  |  |  |  |  |  |  |  |  |  |  |
|  |  |  |  | IFNA2 |  |  |  |  |  |  |  |  |  |  |  |  |  |  |  |  |  |  |  |  |  |  |  |  |
|  |  |  |  | BMPR1B |  |  |  |  |  |  |  |  |  |  |  |  |  |  |  |  |  |  |  |  |  |  |  |  |
|  |  |  |  | IFNA21 |  |  |  |  |  |  |  |  |  |  |  |  |  |  |  |  |  |  |  |  |  |  |  |  |
|  |  |  |  | TNFSF18 |  |  |  |  |  |  |  |  |  |  |  |  |  |  |  |  |  |  |  |  |  |  |  |  |
|  |  |  |  | CCL8 |  |  |  |  |  |  |  |  |  |  |  |  |  |  |  |  |  |  |  |  |  |  |  |  |
|  |  |  |  | IL17RB |  |  |  |  |  |  |  |  |  |  |  |  |  |  |  |  |  |  |  |  |  |  |  |  |
|  |  |  |  | TNFRSF25 |  |  |  |  |  |  |  |  |  |  |  |  |  |  |  |  |  |  |  |  |  |  |  |  |
|  |  |  |  | IL22 |  |  |  |  |  |  |  |  |  |  |  |  |  |  |  |  |  |  |  |  |  |  |  |  |
|  |  |  |  | IL10RB |  |  |  |  |  |  |  |  |  |  |  |  |  |  |  |  |  |  |  |  |  |  |  |  |
|  |  |  |  | IFNAR2 |  |  |  |  |  |  |  |  |  |  |  |  |  |  |  |  |  |  |  |  |  |  |  |  |
|  |  |  |  | CCL18 |  |  |  |  |  |  |  |  |  |  |  |  |  |  |  |  |  |  |  |  |  |  |  |  |
|  |  |  |  | IFNA16 |  |  |  |  |  |  |  |  |  |  |  |  |  |  |  |  |  |  |  |  |  |  |  |  |
|  |  |  |  | CSF2RB |  |  |  |  |  |  |  |  |  |  |  |  |  |  |  |  |  |  |  |  |  |  |  |  |
|  |  |  |  | IL36A |  |  |  |  |  |  |  |  |  |  |  |  |  |  |  |  |  |  |  |  |  |  |  |  |
|  |  |  |  | TNFAIP3 |  |  |  |  |  |  |  |  |  |  |  |  |  |  |  |  |  |  |  |  |  |  |  |  |
|  |  |  |  | IL13RA2 |  |  |  |  |  |  |  |  |  |  |  |  |  |  |  |  |  |  |  |  |  |  |  |  |
|  |  |  |  | IL13RA1 |  |  |  |  |  |  |  |  |  |  |  |  |  |  |  |  |  |  |  |  |  |  |  |  |
|  |  |  |  | CCR9 |  |  |  |  |  |  |  |  |  |  |  |  |  |  |  |  |  |  |  |  |  |  |  |  |
|  |  |  |  | TNFRSF10A |  |  |  |  |  |  |  |  |  |  |  |  |  |  |  |  |  |  |  |  |  |  |  |  |
|  |  |  |  | IFNA7 |  |  |  |  |  |  |  |  |  |  |  |  |  |  |  |  |  |  |  |  |  |  |  |  |
|  |  |  |  | IFNW1 |  |  |  |  |  |  |  |  |  |  |  |  |  |  |  |  |  |  |  |  |  |  |  |  |
|  |  |  |  | XCL2 |  |  |  |  |  |  |  |  |  |  |  |  |  |  |  |  |  |  |  |  |  |  |  |  |
|  |  |  |  | TNFSF14 |  |  |  |  |  |  |  |  |  |  |  |  |  |  |  |  |  |  |  |  |  |  |  |  |
|  |  |  |  | CCR2 |  |  |  |  |  |  |  |  |  |  |  |  |  |  |  |  |  |  |  |  |  |  |  |  |
|  |  |  |  | BMP15 |  |  |  |  |  |  |  |  |  |  |  |  |  |  |  |  |  |  |  |  |  |  |  |  |
|  |  |  |  | BMP10 |  |  |  |  |  |  |  |  |  |  |  |  |  |  |  |  |  |  |  |  |  |  |  |  |
|  |  |  |  | CCL15-CCL14 | |  |  |  |  |  |  |  |  |  |  |  |  |  |  |  |  |  |  |  |  |  |  |  |
|  |  |  |  | TGFBR1 |  |  |  |  |  |  |  |  |  |  |  |  |  |  |  |  |  |  |  |  |  |  |  |  |
|  |  |  |  | IFNA5 |  |  |  |  |  |  |  |  |  |  |  |  |  |  |  |  |  |  |  |  |  |  |  |  |
|  |  |  |  | BMP7 |  |  |  |  |  |  |  |  |  |  |  |  |  |  |  |  |  |  |  |  |  |  |  |  |
|  |  |  |  | IFNA14 |  |  |  |  |  |  |  |  |  |  |  |  |  |  |  |  |  |  |  |  |  |  |  |  |
|  |  |  |  | IL20RB |  |  |  |  |  |  |  |  |  |  |  |  |  |  |  |  |  |  |  |  |  |  |  |  |
|  |  |  |  | IL10RA |  |  |  |  |  |  |  |  |  |  |  |  |  |  |  |  |  |  |  |  |  |  |  |  |
|  |  |  |  | IFNA17 |  |  |  |  |  |  |  |  |  |  |  |  |  |  |  |  |  |  |  |  |  |  |  |  |
|  |  |  |  | CCR6 |  |  |  |  |  |  |  |  |  |  |  |  |  |  |  |  |  |  |  |  |  |  |  |  |
|  |  |  |  | TGFB3 |  |  |  |  |  |  |  |  |  |  |  |  |  |  |  |  |  |  |  |  |  |  |  |  |
|  |  |  |  | CCL15 |  |  |  |  |  |  |  |  |  |  |  |  |  |  |  |  |  |  |  |  |  |  |  |  |
|  |  |  |  | CCL4 |  |  |  |  |  |  |  |  |  |  |  |  |  |  |  |  |  |  |  |  |  |  |  |  |
|  |  |  |  | CCL27 |  |  |  |  |  |  |  |  |  |  |  |  |  |  |  |  |  |  |  |  |  |  |  |  |
|  |  |  |  | TNFRSF13B |  |  |  |  |  |  |  |  |  |  |  |  |  |  |  |  |  |  |  |  |  |  |  |  |
